# A New Approach for Inspection of Selected Geometric Parameters of a Railway Track Using Image-Based Point Clouds

**DOI:** 10.3390/s18030791

**Published:** 2018-03-06

**Authors:** Grzegorz Gabara, Piotr Sawicki

**Affiliations:** Institute of Geodesy, University of Warmia and Mazury in Olsztyn, 10-719 Olsztyn, Poland

**Keywords:** DSLR camera, dense matching, point clouds, 3D object reconstruction, track geometry

## Abstract

The paper presents the results of testing a proposed image-based point clouds measuring method for geometric parameters determination of a railway track. The study was performed based on a configuration of digital images and reference control network. A DSLR (digital Single-Lens-Reflex) Nikon D5100 camera was used to acquire six digital images of the tested section of railway tracks. The dense point clouds and the 3D mesh model were generated with the use of two software systems, RealityCapture and PhotoScan, which have implemented different matching and 3D object reconstruction techniques: Multi-View Stereo and Semi-Global Matching, respectively. The study found that both applications could generate appropriate 3D models. Final meshes of 3D models were filtered with the MeshLab software. The CloudCompare application was used to determine the track gauge and cant for defined cross-sections, and the results obtained from point clouds by dense image matching techniques were compared with results of direct geodetic measurements. The obtained RMS difference in the horizontal (gauge) and vertical (cant) plane was RMS∆ < 0.45 mm. The achieved accuracy meets the accuracy condition of measurements and inspection of the rail tracks (error m < 1 mm), specified in the Polish branch railway instruction Id-14 (D-75) and the European technical norm EN 13848-4:2011.

## 1. Introduction

Non-invasive 3D measurement techniques in the railway sector use active and passive measurement methods. In this scope of survey and inspections, mainly mobile systems are used, based on terrestrial laser scanning (TLS), which is often supported by sequences of digital images captured by video cameras (e.g., Lynx Mobile Mapper by Teledyne Optech Vaughan, Canada). Mobile LiDAR data were the basic data source for railway line surveys [1], for automated detecting and modelling of rails [2], in automated recognition of railroad infrastructure [3], in automatic extraction of centerlines of railroads [4] and for measuring the railway clearance gauge [5].

However, vision-based methods using digital sensors and data processing were applied among others for defect detection on rail surfaces [6], for measurement of railroad clearance obstacles [7], for continuous monitoring and assessment of the condition of the rail tracks [8], as well as for automated visual inspection of railroad tracks [9].

The photogrammetric reconstruction of 3D objects is currently performed based on point clouds generated by dense image matching techniques. One of the most commonly implemented methods is the SfM (Structure from Motion) technique with extensions [10]. The dense point clouds and advanced data processing allow to recognize and completely reconstruct 3D objects, and then to measure and extract geometric and semantic information [11]. The image-based point clouds measurement method may be used to measure shapes [12], displacements and deformations of objects in static [13], dynamic [14], and multi-temporal processes [15]. 

An important task and research problem is testing the accuracy of point clouds generation from dense image matching and data processing for 3D surface reconstruction in close range and searching for new areas of practical implementation of that method. This task was initially realized in our previous work [16]. In this paper, we extend it by the following: the result of proposed photos configuration, the results of study on 3D point clouds accuracy, the visualization and analytical analysis of quality and accuracy of railway track 3D models, discussion of the obtained results in relation to technical standards, the scheme of a proposed concept of a mobile vision system, and the final results of testing proposed image-based point clouds measuring method.

A new possible application is proposed by the novel approach to the use of point clouds and mesh models generated from sequences of digital images for 3D reconstruction and measurement of selected geometric parameters of railway tracks with high accuracy (error m < 1 mm). According to the authors’ knowledge, a mobile vision system and other scientific works that only use imaged-based point clouds as a measuring method for railway purposes and allow to achieve such high accuracy, do not exist at present. 

## 2. Geometric Parameters of a Railway Track

**Figure 1:**
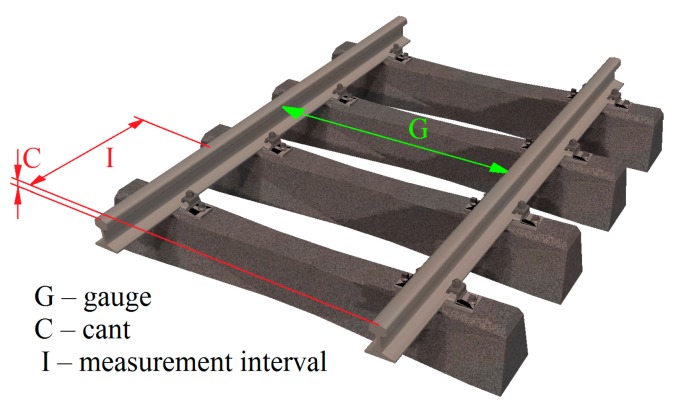
Graphic definition of the Cant (C), Gauge (G), and measurement interval (I).

Railway tracks are 3D elongated objects which consist of elements with cross-sections similar to the Double T-Bar (DTB). At present, most of the complete measurements of geometric parameters of railway tracks are performed using a track geometry measuring trolley (TGMT) [17,18,19,20], coupled with a one-man station. For rectilinear sections of tracks, the system measures standard geometric components (Figure 1): the spatial location of tracks, the track gauge (G), and the cant (C), also named cross level or super elevation. The track gauge is a slope distance measured between the rail heads, typically 14 mm below the running surface, and the cant is the difference of elevation between the rail heads [21,22]. Measurements of geometric parameters are performed for a defined interval (I) of the track distance [22,23]. According to the Polish branch railway instruction Id-14 (D-75) [22], the measurement of rail track gauge and cant is done every 5 m on a straight line section and every 2.5 m in a curve length with a radius less than 300 m, but for the computation of other geometric parameters (twist, gauge gradient) a 1.0 m measuring interval is needed. In addition, the guard check gauge and the guard face gauge are measured for turnouts. Examples of mobile measurement platforms include the Trimble GEDO CE [24,25] and the Topcon GG-05 [25] systems. Geometric parameters of tracks may be also measured using a hand track gauge and a cant measuring device MOD (manually operated devices) [26], e.g., Graw DTG Track or Sola SW 9182.

## 3. Acquisition, Measurement and Processing of Digital Images Set

The presented study was performed on the basis of configuration of terrestrial photos and the reference control network, which had exactly simulated the conditions of operations of the concept of a mobile vision platform for measuring selected geometric parameters of a railway track. 

### 3.1. Test Object

The test object was the rectilinear section of a railway track 1 m in length. The dataset adopted for testing includes the signalized control points and uncalibrated imagery for generation of photogrammetric dense point clouds.

Artificially signalized control points (CrlP) were placed on each rail head in 3-1-3 groups. A set of CrlP simulated approximately the referential control points template (constant, fixed). The distances between control points were measured in all possible combinations using the caliper ACCUD ABS 118-080-11 (2 m in length) of the practical reading accuracy S_D_ = 0.05 mm. Then, the free adjustment of linear network was performed using the WinKalk application [27], and a mean planar error for 14 control points S_XY_ = 0.22 mm was obtained. Elevations of control points were determined using precise levelling (PL) with the accuracy S_Z_ = 0.10 mm (Leica DNA03) [28]. A geodetically measured control points set was applied for point clouds orientation and calibration.

At the initial stage of research, many different configurations of multiple photos were tested (for example [16]) to get the best results of image-based point clouds generation in commonly used software systems, and only the best one is presented in this paper. The following selection criteria of the optimization were assumed: the smallest number of applied photos, deviation on control points, and the quality of generated point clouds for the 3D rail reconstruction. The authors chose the configuration of six photos (nearly vertical, oblique, and oblique–convergent photos). 

**Figure 2:**
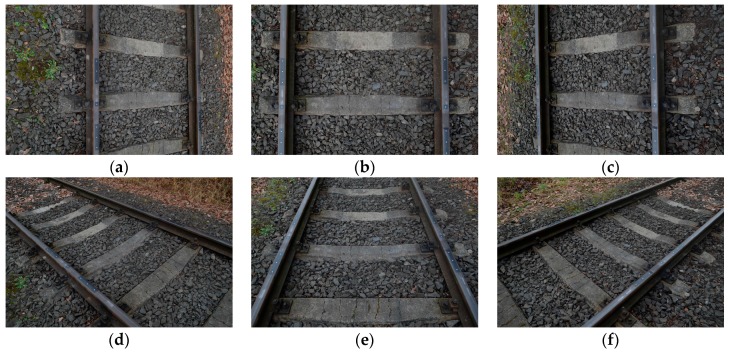
Configuration of six terrestrial photos of the tested section of the track: nearly vertical photo (**b**), oblique photos (**a**,**c**,**e**), and oblique–convergent photos (**d**,**f**).

The digital SLR (Single-Lens-Reflex) Nikon D5100 camera, equipped with a CMOS (Complementary Metal-Oxide Semiconductor) sensor (23.6 × 15.6 mm size, resolution 4928 × 3264, pixel size p’_xy_ = 4.8 μm) and a lens with optical stabilization [29], focused (focal f = 18 mm) on imaging distance Y_F_ = 2.0 m, was used to acquire the photos (Figure 2).

Performed research works were characterized by the following real conditions of photogrammetric acquisition and processing of digital images:

- long-shaped object,
- six photos in two strips,
- short focal length of the wide-angle camera lens,
- full longitudinal overlap,
- base/distance ratio υ = ½ ÷ 1,
- use of a reference system with 14 control points (S_XYZ_ = 0.24 mm),
- self-calibration of DSLR Nikon D5100 camera 16 MP (megapixels),
- images radiometric quality dependent on weather (dry and wet rails) and lighting conditions.

Depending on the local scale of imaging [30], the diameter of signalized control points on the images varied from 7 to 21 pixels. The signalized control points were measured using the center weighted method (centroid operator) by means of Matching, the original software [31]. The calculated mean subpixel accuracy of this automatic measurement was s_x’y’_ = 0.15 pixel.

### 3.2. Point Clouds and Mesh Generation

The practical verification of the quality and reliability of the 3D rail track models generation was performed using two methods implemented in two different software:

- the Scalable Multi-View Stereo (MVS) in RealityCapture v. 1.0.2.2256 RC (commercial license) of Capturing Reality s.r.o [32,33,34],
- the modified stereo Semi-Global Matching (SGM) in PhotoScan v. 1.2.6 (commercial license) of Agisoft LLC [12,35,36].

The RealityCapture is quite a new application on the market, but it has enjoyed very good opinions among the users, and PhotoScan is a well-known and very popular suite. We tested them with some other application before [16,37] and after that we decided to use them for the verification of our measuring method. Both applications are dedicated, first of all, to reconstruction of 3D objects based on a large number of variously oriented digital images. They also allow an automatic determination of camera interior orientation parameters, the elements of a spatial orientation of photos, generation of the dense point clouds, a 3D mesh model, and finally the DSM (Digital Surface Model) and a orthomosaic.

The Agisoft PhotoScan was used by authors as an additional computation and research tool for verification of digital processing results in RealityCapture. This methodology allows to receive some additional information about applications reliability and functionalities in the field of a railway optical metrology, but a benchmark is not the main focus of the paper.

The selection of functions, configuration, and modification of data processing options are performed in the interactive and manual mode in the RealityCapture and PhotoScan software. Different functionalities of both tools required different methodology of the advanced digital processing. In both cases, processing parameters were set using the option without *scaling images* and with an unlimited number of *features objects* (“Max. features objects” option), which means that the computations are longer and applications find as many key points as possible, but with a bigger number of less reliable points. They should be rejected at the tie point estimation stage [38]. 

Possibly, higher accuracy and automation of processing can be achieved when structured light is used for projection of artificial profiles on the rail track (interested area is unambiguously defined). That should ensure the optimum exposure parameters, independently of external illumination conditions.

Therefore, the computations of the 3D rail track models were performed in two variants of dense point clouds generation:

- Basic model (BM) processed without an externally defined profile on the track (Section 4.1),
- Profile model (PM) performed with an externally defined (in situ) artificial profile that simulated structured light on the track (Section 4.2).

**Figure 3:**
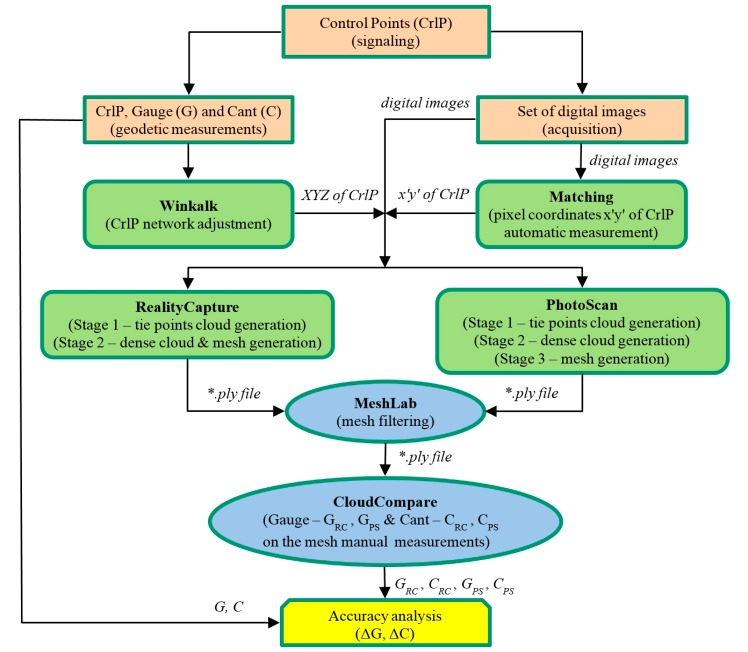
Measurement and processing workflow in the tests.

The study workflow of geodetic and photogrammetric measurements and processing is presented in the Figure 3.

#### 3.2.1. RealityCapture Settings

Image matching, construction, and coloring of a model are required to generate a mesh draped on the point cloud in the RealityCapture [37]. Image matching was performed using the “Alignment” option. *Detector sensitivity* was set to “Ultra”, *max. features per megapixel* was set at 800 thousands, *max. features per image* was set at 3 million, *max. feature reprojection error* was set at 2.0 pixels, *images overlap* was set to “High” and *image downscale factor* was set at 1.0. The *minimal distance between two vertices* was d = 0.1 mm. The camera calibration model was selected by defining the number and approximate values of parameters to be determined (f, x’_o_, y’_o_, K_1_ ÷ K_3_, P_1_, P_2_, C_1_, C_2_). The construction of the model was performed according to the “Normal” variant with manual setting of parameters, identical to parameters in the “High detail” option. This operation allowed to omit errors of the CUDA drivers of the old workstation graphic card, but after RealityCapture update, the problem does not appear any longer (older CUDA graphics card are also supported). During model generation: in image depth map calculation and model colorize *image downscale* was set at 1.0 and *coloring method* was set to multi-band.

#### 3.2.2. PhotoScan Settings

When the PhotoScan application is used to generate a 3D model, image matching, generation of a dense point cloud, construction of a model, and texture building must be performed by several steps, as shown in numerous papers [30,36]. For these purposes, the *processing accuracy* was set as “Highest” with searching for tie points for each possible stereo-pair (option “*Pair preselection*: disabled”). The *key point limit* was set at 3 million and *tie points limit* was 800 thousands. The dense point cloud was generated at ”Ultra high” settings with *aggressive depth filtering*. The construction of the mesh model was performed using the surface type “Arbitrary” and the *face count* “High”, without interpolation. Texture building was performed with the *mapping model* “Generic” and “Average” *blending mode*, which means that PhotoScan tries to create as uniform texture as possible for arbitrary geometry and uses the weighted average value of all pixels from individual photos for texturing [38] (p. 18).

### 3.3. Mesh Filtering and Measurement

The 3D mesh models of the test section of railway tracks, generated from dense point clouds with the use of RealityCapture and PhotoScan software, were further processed using MeshLab [39,40]. Model filtration consisted of the elimination of mesh edges longer than 2 cm and the elimination of surrounding areas of the test section. The final section of the railway track model was limited to the width of the railway sleeper and to the distance between three successive sleepers.

**Figure 4:**
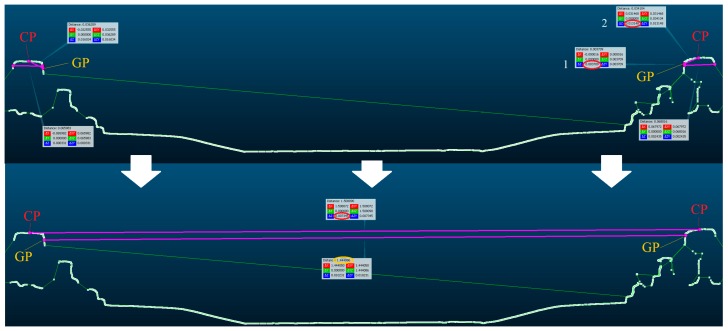
Cant point (CP) and gauge point (GP) definition and measurement of the track gauge and cant in CloudCompare application.

The track gauge and cant values were determined for the railway track section located in the center of the test section using CloudCompare [41]. Within the area where control points were located, for the section d = 1.0 m a tracing line was marked along the rail and cross-sections were created with the interval Δd = 0.1 m. The interested area of the cross-sections were the cant points (CP), located in the center of the rail heads and the gauge points (GP) defined at the side of the rail heads, 15 mm below the CP point. The scheme of determination of CP and GP points is presented in Figure 4. First, CP points and then GP points were determined.

The closest point to the center of rail on the rail head and the GP point localization were determined manually by the user. Differences of elevation between the CP points were the cant value. The gauge value was analytically calculated as a distance between GP points. The measured gauge and cant values were compared with the results of direct geodetic measurements.

## 4. Results of the 3D Model Processing

RealityCapture and Agisoft PhotoScan applications were used for processing on the workstation with the processor Intel^®^ Core™ i7-950 (8 MB Cache, 3.06 GHz), 24 GB RAM DDR3-1333 MHz memory, NVIDIA^®^ Quadro^®^ 4000 graphic card and SATA3 7200 rpm disk.

### 4.1. Basic Model Analysis

Table 1 presents parameters of processing of generated point clouds and 3D mesh models. Calculated RMS values are related to deviations on control points of the 3D model.

**Figure 5:**
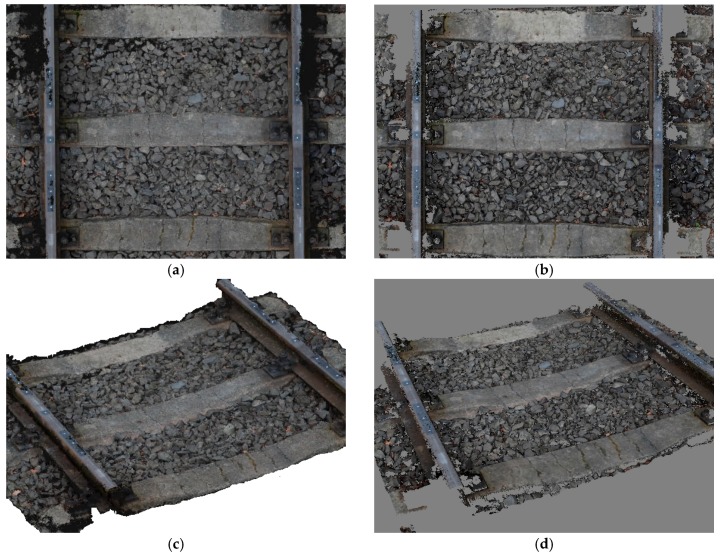
Generated 3D mesh model of the railway track (top and solid view) in Reality Capture (**a**,**c**) and PhotoScan (**b**,**d**) software.

**Figure 6:**
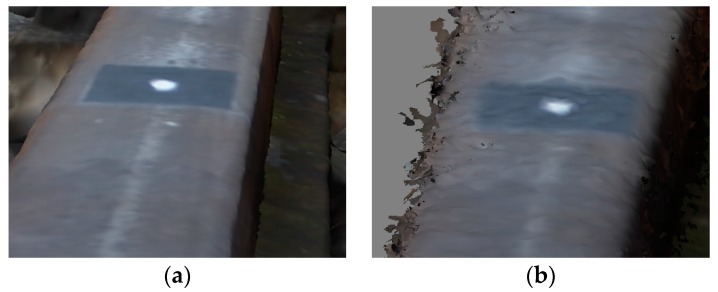
Fragment of generated 3D mesh model of, e.g., left rail (solid view) in RealityCapture (**a**) and PhotoScan (**b**) software.

Visualizations of 3D mesh models, generated with the use of MVS and SGM techniques implemented in RealityCapture and PhotoScan applications, respectively, are presented in Figure 5 and Figure 6. 

The processing time of a railway track reconstruction, in the case of RealityCapture, was shorter. In the authors’ opinion, it is related to algorithms which are used by applications. PhotoScan uses pairwise matching [38], while RealityCapture uses loop-closing techniques based on SURF-based visual words [42] and tf-idf scores [43]. Furthermore, in the RealityCapture most of the computations are performed by GPU cores, while PhotoScan uses CPU and random-access memory (RAM) and only some calculations are performed by GPU.

**Figure 7:**
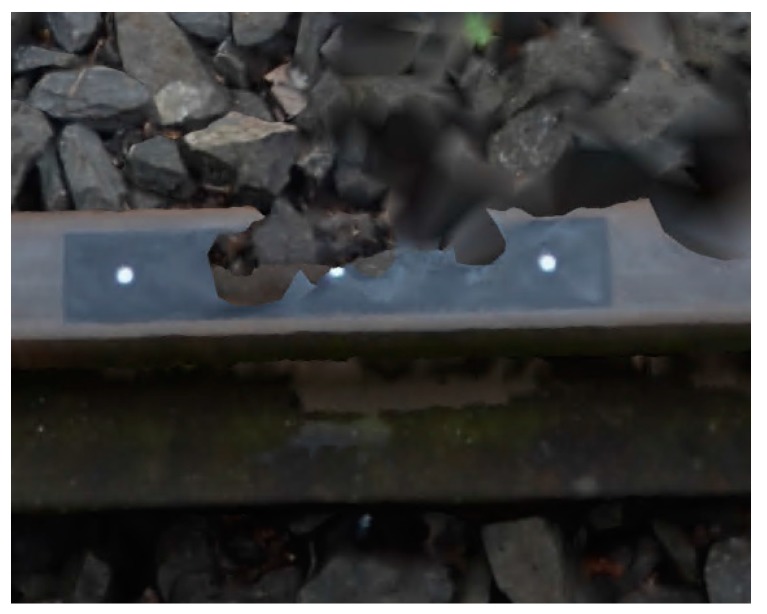
Holes on control points (CrlP) triplet in the 3D mesh model generated by RealityCapture software.

The 3D surface reconstruction obtained using RealityCapture application is of better visual quality, with the exclusion of model noise in fragments with the control points (CrlP) triplet. They were apparently caused by the amorphous texture of the signalized control points background (Figure 7). 

**Figure 8:**
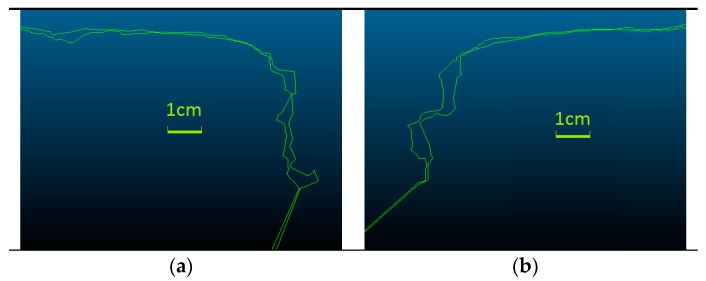
Left (**a**) and right (**b**) rail double edges on one cross-section generated by PhotoScan.

PhotoScan generated a denser point cloud, based on a smaller amount of tie points. The use of the processing parameters in the “highest quality” option does not eliminate defects on 3D mesh model. The measurements on the model were performed using CloudCompare application. During the analysis of the 3D model generated using PhotoScan, double edges within an approximate distance of 3 mm (Figure 8) on rail head were observed.

Table 2 presents the track gauge (G) and cant values (C), directly measured in the field using a caliper and precise levelling (PL), values measured (CloudCompare) on the 3D models generated in RealityCapture (G_RC_, C_RC_) and PhotoScan (G_PS_, C_PS_) software and additionally their differences (∆G_RC_, ∆C_RC_, ∆G_PS_, ∆C_PS_). On the section of a railway track (1 m in length), the cross-sections in the interval of 0.1 m is defined, which does not coincide with points used for global geo-referencing the 3D reconstruction (excluding CrlP 1 and 7). 

### 4.2. Profile Model Analysis

Due to visual analysis and the time consumed to process BMs, the MVS algorithm (RealityCapture) appears to provide more stable computation, and in this part of study it was the only algorithm used. In the case of the PM computation, the parameters and results of digital processing were almost identical to BM and they are shown in Table 1. 

**Figure 9:**
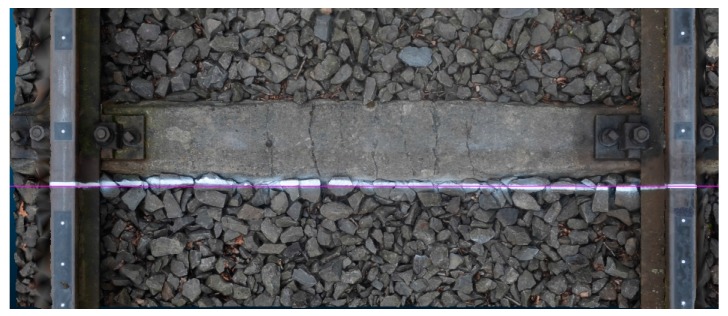
Generated 3D model of railway track with simulated structured light (SSL) profile.

Figure 9 presents a 3D extended model of the railway track with the simulated structured light (SSL) profile. It also shows how the SSL profile coincides with the computed cross-section that is perpendicular to the rail.

**Figure 10:**
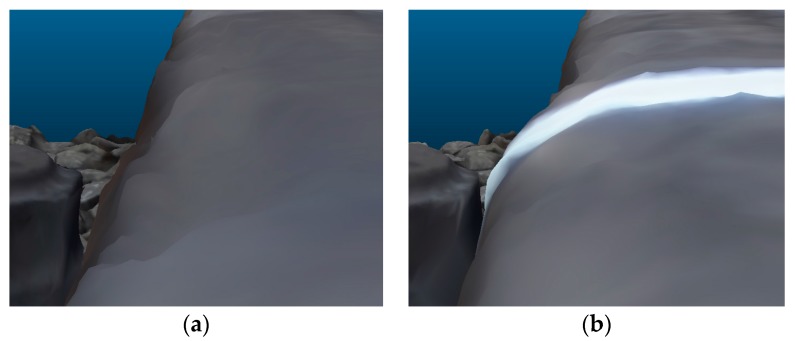
Right rail track 3D model without (**a**) and with (**b**) SSL profile.

**Figure 11:**
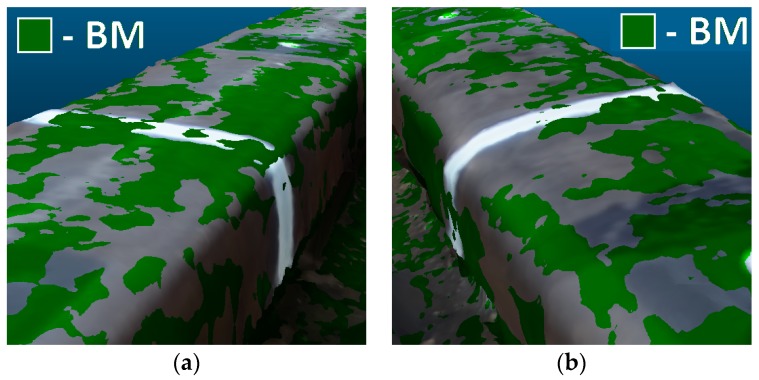
Penetrate maps of the 3D models: basic model (BM; green color) in relation to the profile model (PM) (**a**) on the left; (**b**) on the right rail.

In the next step of the two models, the BM and PM were compared (Figure 10 and Figure 11). The question is analyzed in more detail in Figure 11, where both 3D models are superimposed. Places where fragments of BM cover PM are marked by green color. 

**Figure 12:**
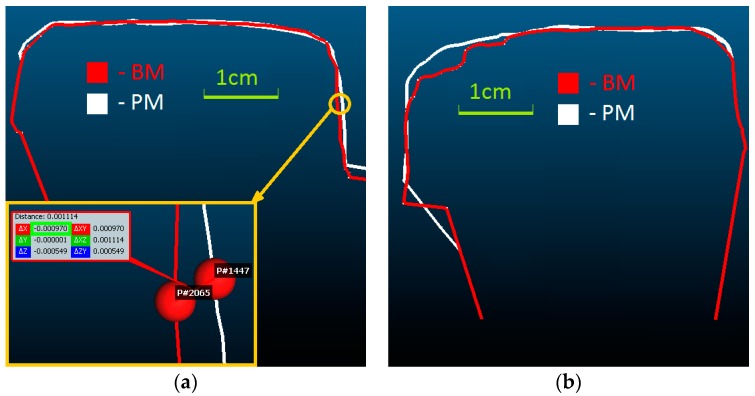
Discrepancies between cross-sections on the 3D models (BM and PM) (**a**) on the left; (**b**) on the right rail.

In places on BM where the running surface is generated with some deformations, a refinement of the rail heads edges on PM could be observed (Figure 12). The measured distances on the gauge points between 3D models are Δ_GP (L)_ ≈ 1.0 mm for left rail and Δ_GP (R)_ ≈ 0.1 mm for right rail, and the Δ_CP_ ≤ 0.3 mm for running surfaces.

Track gauge and cant measurements on 3D models compared to geodetic survey are presented in Table 3.

The obtained RMS differences of the BM and PM geometric parameters in the horizontal (gauge G) and vertical (cant C) planes in relation to geodetic measurements using the caliper and precise levelling amount respectively to RMS∆_BM_ = 0.38 mm and RMS∆_PM_ = 0.48 mm. The values of these differences are statistically insignificant.

## 5. Discussion

The experimental study of the proposed concept of image-based point clouds measuring method for geometric parameters determination of a railway track was performed on the basis of the photogrammetric configuration of six terrestrial photos (nearly vertical, oblique, and oblique–convergent photos). In these conditions, the measurement potential of 3D object reconstruction in close range using photogrammetrically generated point clouds was also tested. 

Two methods implemented respectively in RealityCapture and PhotoScan applications were used to generate the dense point cloud and the 3D mesh models for the test section of the railway track. The study found that the appropriate 3D models can be generated using point clouds from dense image matching. In addition, a discussion of the results of processing and measurements using both applications is presented. The processing results proved the higher usefulness of the RealityCapture application in railway purposes, which generated the 3D model faster, of higher visual quality and robust.

The comparison of the basic model and the model performed with a simulated structured light profile on the track shows that both modes can be used in geometric parameters of the track computation. The main difference is the independence from lighting and reflection conditions in case of using structured light for artificial profile definition.

The test results of the track gauge and cant values obtained using the image-based point clouds measuring method were compared with the results of direct precise geodetic measurements. The obtained RMS difference in horizontal (gauge) and vertical (cant) plane amounted to RMS∆ < 0.45 mm, which is significantly lower than allowable values defined in the European technical norm EN 13848-4:2011 (track gauge ±1 mm, cant ±2.5 mm) [23]. In relation to the tolerances (track gauge and cant ±2 mm) of a TGMT system that was used to measure the Zürich-Thalwil Tunnel rails where the projected operational speed was 200 km/h [44] (p. 141), our proposed measuring method has fulfilled these conditions. Furthermore, in comparison [45] to other geodetic measurements using some fixed-points, chord measuring systems, and inertial measuring systems, described in [46], the obtained results using photogrammetric method are satisfactory. Besides this, it is compliant with EN 13848 series [21,23,45,47,48]. Basing on the track quality class (TQC) and track quality index (TQI) parameters [48], which depend on a speed function and vehicle ride quality, the proposed measuring method can be used for the evaluation of 5th class track geometry quality.

**Figure 13:**
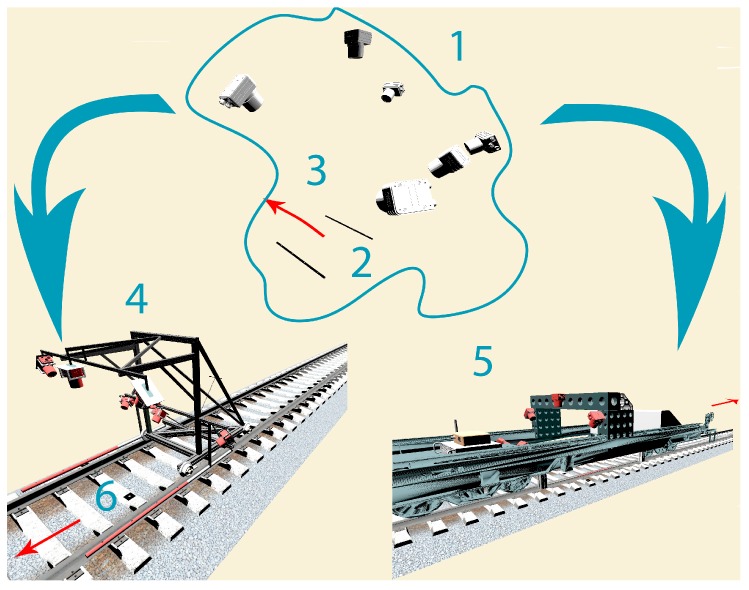
Scheme of proposed image-based method application for railway track inspection. (1) measurement environment (configuration of six cameras and reference template); (2) reference template; (3) arrow shows measurement direction; (4) concept of measuring trolley (TGMT) with implemented method; (5) concept of measuring platform with implemented method; (6) possible placement of control points for geo-localization.

The first positive experimental results of the presented novel approach encourage the authors to continue work on the construction of a dedicated, low-cost mobile vision measuring system. The proposed designs of implementation our method are presented in Figure 13. The same figure shows also two possibilities of use our measuring method: as a mobile trolley and as a railway platform. Both of them use reference templates for computation of a model true geometry. The possible placement and outlook of control network is included (six on Figure 13), but it is needed only for transformation from local railway Coordinate System (millage) to National Coordinate System or ETRF2000. Because track inspection measurements are geolocalized by millage, the control network is mentioned as an extension, e.g., for drawing maps or for future railway lines modernization. The standard localization of control network is two points by each 500 m, which will be enough for this purpose.

## 6. Conclusions

The presented results concern the testing of the measuring method using point clouds from dense image matching applied to the 3D reconstruction and measurements of the selected geometric parameters (gauge and cant) of a railway track. The performed tests have initiated the design of a mobile vision measuring platform. This concept represents an alternative to common railway measuring systems. A continuously working system could be in practice a competitive solution for the currently used manually operated devices (MOD) [26] and expensive geodetic mobile measurement trolleys (TGMT) [25,44], dynamic inertial measuring systems [49], and other vehicle reaction measuring systems [46]. Besides, the use of a photogrammetric method enables faster track geometry irregularities detection and acquisition of numeric and semantic big data of the railway and its surrounding area. 

The achieved accuracy meets the accuracy condition of measurements and inspection of railway tracks (error m < 1 mm), specified in the Polish branch railway instruction Id-14 (D-75) [22] and the European technical norm EN 13848-4:2011 [23]. 

## Figures and Tables

**Table 1:** Parameters and results of images processing.

Processing Parameters & Results	RealityCapture	PhotoScan
Processing time	22 min 20 s	327 min 2 s
Point cloud coverage area	6.99 m^2^	6.99 m^2^
No. of used tie points	84,728	59,527
No. of cloud points	5,147,830	25,980,265
Point density per m^2^	1,739,132	3,717,550
Model coverage area	2.96 m^2^	2.96 m^2^
No. of model faces	10,083,505	3,078,302
No. of model vertices	5,063,102	2,321,412
StDev s_x’y’_ on CrlP	0.59 pix	0.39 pix
RMSΔ_XY_ Dev on CrlP	0.213 mm	0.280 mm
RMSΔ_Z_ Dev on CrlP	0.075 mm	0.048 mm
RMSΔ_XYZ_ Dev on CrlP	0.226 mm	0.284 mm

**Table 2:** Track gauge (G) and cant (C) measurements results on the 3D rail track basic model.

No. of Cross-Section	Measurement (mm)						Measurements Differences (mm)			
	Caliper	PL	RealityCapture		PhotoScan		RealityCapture		PhotoScan	
	Gauge (G)	Cant (C)	Gauge (G_RC_)	Cant (C_RC_)	Gauge (G_PS_)	Cant (C_PS_)	ΔG_RC_ = G − G_RC_	ΔC_RC_ = C − C_RC_	ΔG_PS_ = G − G_PS_	ΔC_PS_ = C − C_PS_
1	1444.01	7.19	1444.6	7.1	1444.61	7.6	−0.59	0.09	−0.60	−0.41
2	1444.01	7.19	1443.63	7.6	2 edges	2 edges	0.38	−0.41	-	SSSS-
3	1444.00	7.18	1444.66	7.5	1442.19	7.6	−0.66	−0.32	1.81	−0.42
4	1444.00	7.17	1444.12	7.1	1443.68	7.5	−0.12	0.07	0.32	−0.33
5	1444.00	7.16	1444.09	7.3	1441.39	7.3	−0.09	−0.14	2.61	−0.14
6	1443.99	7.16	1444.65	7.5	1443.88	6.9	−0.66	−0.34	0.11	0.26
7	1443.99	7.15	1444.53	7.4	1443.68	7.0	−0.54	−0.25	0.31	0.15
8	1443.99	7.16	1444.36	7.5	1442.83	6.9	−0.37	−0.34	1.16	0.26
9	1444.00	7.16	1443.97	6.9	1443.72	6.8	0.03	0.26	0.28	0.36
10	1444.00	7.17	1443.91	7.4	1442.28	6.9	0.09	−0.23	1.72	0.27
				RMSΔG_RC_C_RC_,ΔG_PS_C_PS_			0.43	0.27	1.29	0.30

**Table 3:** Track gauge (G) and cant (C) measurements results on the 3D rail track models.

Measurements (mm)						Differences (mm)			
Caliper	PL	Basic Model (BM)		Profile Model (PM)					
Gauge (G)	Cant (C)	Gauge (G_BM_)	Cant (C_BM_)	Gauge (G_PM_)	Cant (C_PM_)	ΔG_BM_ = G − G_BM_	ΔC_BM_ = C − C_BM_	ΔG_PM_ = G − G_PM_	ΔC_PM_ = C − C_PM_
1444.00	7.18	1444.50	7.38	1443.74	7.80	−0.50	−0.20	0.26	−0.62
